# The Amending Bill

**Published:** 1913-07-05

**Authors:** 


### NATIONAL INSURANCE ACT DEPARTMENT.

### LIX.—The Amending Bill.

The text of the amending Bill to the National Insurance Act, which was introduced into Parliament by the Chancellor of the Exchequer on Tuesday, June 24, has now been published. Its provisions will well repay careful consideration by all who are interested in questions of national health and well-being, and we take the earliest opportunity of bringing to the notice of our readers the nature of the proposals set forth.

The Bill is not pretentious in its scope, for it consists of thirteen comparatively short clauses only, and contains but one schedule, and is thus very far removed in point of magnitude and importance from the principal Act, which contains 115 sections and no less than nine comprehensive schedules.

In his introductory speech Mr. Lloyd George indicated that no proposal was made which would touch the main structure of the National Insurance Act, and gave as his reason that as only six months' experience of that Act had been gained it was too early to come to any conclusions with regard to its main outlines. It remains to be seen whether Parliament will allow the measure to proceed without taking further cognisance of the numerous amendments that have been suggested as the result of experience, short though it has been. Unless further amending clauses be added it would seem that other Bills will be required in the near future to make good the deficiencies in the proposals.

### Additional Parliamentary Grants.

The real reason for the introduction of the present Bill is apparently contained in Clause 1, which is designed to redeem the pledge given to Parliament by the Prime Minister that he would obtain legislative sanction for the additional expenditure incurred in connection with the provision of medical benefit as a result of the negotiations between the Chancellor of the Exchequer and the medical profession. Had it not been for this pledge it is more than probable that the proposals now put forward would have had to wait, along with other suggested amendments, until a more convenient season.

It is now proposed to regularise the additional payment to the doctors, but as this sum is entirely provided by means of a Government grant, and not out of the contributions paid by, or on behalf of, the insured persons, the clause is so framed as to limit its amount to such a sum as Parliament may from time to time determine.

Thus it would appear that the remuneration of the doctors is to remain at the mercy of the annual vote in Parliament in the Finance Act.

The second sub-clause, however, proposes to give the advantage of the additional grant to the doctors in respect of those persons who on account of old age or permanent disablement do not become insured under the Act, and who were members of friendly societies when the principal Act was passed.

We pointed out in a recent issue that the dispute between the Chancellor and the medical profession had led to the resignation of club practice by large numbers of doctors, with the result that on the completion of the negotiations the doctors were unwilling to attend club patients except on the terms arranged with the Chancellor. This meant, almost without exception, a serious drain on the funds of the friendly societies in order to provide the higher cost of medical treatment and attendance for those members who remain in the voluntary section, but who did not become insured persons under the provisions of the Act.

### Medical Benefit for Old Members.

It is proposed that the societies with members such as are here described shall not continue to be under the same disability as at present, so far as those members are concerned who belonged to the societies at the passing of the principal Act on December 16, 1911. The disability will not be entirely removed in all cases, for it is the additional grant only which is to be applied towards the payment for medical treatment of such members, whereas in point of fact the extra charge on the society entailed by the new agreement with the club doctor is frequently considerably greater than the amount of the additional Government grant.

The proposal should, however, give general satisfaction to the societies, as it is a clear admission that their position has been adversely affected by the National Insurance Act, and it is at least a step towards the rectification of that anomaly.

It will be remembered that the additional Government grant is equivalent to 2s. 6d. per member per annum, and such a sum is, of course, of considerable moment to a society with a number of old members.

### Trades Unions also to be Benefited.

The Chancellor of the Exchequer was asked whether the amendment would apply to trades unions as well as to friendly societies, and replied that it would apply to all approved societies.

At first it would seem from the text of Clause 1 (2) of the amending Bill that provision has only been made in this respect for the friendly societies, and not for the trades unions. The words referred to in the principal Act (Section 15 (2) (*e*)) distinctly limit the operation to a friendly society which, or a separate section of which, becomes an approved society.

Clause 5 (2) of the amending Bill, however, makes it clear that the advantages of Section 15 (2) (*e*) are to extend not only to friendly societies, but also to all classes of societies that have been approved by the Insurance Commissioners. The separation of Clause 5 (2) from Clause 1 was presumably deemed to be necessary from a technical point of view for drafting purposes, but it appears to us to be much more appropriate if inserted as a further sub-clause to Clause 1.

The only apparent reason for its inclusion in Clause 5 is that that clause deals with another aspect of medical benefit.

### The Income Limit for Voluntary Contributors.

Here, again, the demand of the doctors is in evidence, and a promise made by the Chancellor to the profession is redeemed by its insertion in the Bill. The Chancellor had promised to insert a provision in the first amending Bill which should impose an income limit of £160 per annum for voluntary contributors as the maximum which, if exceeded, would disqualify a voluntary contributor from the receipt of medical benefit. There was really no urgency for such provision to be made, for there cannot possibly be a voluntary contributor under the Act with an income of more than £160 per annum until the National Insurance Act has been five years in operation.

As an offset to the loss of medical benefit by the insured person in the circumstances indicated it is proposed that the weekly contribution for a voluntary contributor who has an income of more than £160 per annum shall be 1d. less than would otherwise have been payable by him. It will be noticed in this connection that apart from this minor concession the question of an income limit for medical benefit is left untouched. There must be many cases where manual labourers are earning more than £160 a year, and are therefore insured as employed contributors, and their right to medical benefit will continue in spite of the views of the doctors.

The general result of the new proposals regarding medical benefit will therefore be that Parliament will have full statutory authority for its yearly grants to the medical profession; the friendly societies and trades unions that have become approved societies will obtain relief in respect of the additional cost of providing the benefit to old members; and the doctors will not be called upon to attend persons on Insurance Act terms when they are voluntary contributors with an income from all sources of more than £160 per annum.

### The Possibility of Further Grants.

It should be mentioned in passing that the proposal to obtain statutory authority for the additional Government grant for medical benefit is contained in a clause which is so drafted as to enable similar grants to be made at the discretion of Parliament on account of any of the benefits conferred under the health insurance provisions of the Act, or for the expenses incurred in their administration. This, of course, opens a wide field of possibilities, for although the Government may have no immediate intention of calling upon Parliament for additional funds in connection with National Insurance, there is at least a possibility that the insistent demand of a considerable section of the approved societies for larger funds for administrative expenses may require them to take action in the not distant future.

### Alterations Affecting Sickness Benefit.

So far we have only dealt with Clauses 1 and 5, which principally refer to medical benefit. Space will only allow brief mention of the provisions of Clause 2, and we must defer until next week the consideration of further clauses.

Clause 2 proposes to amend the provision of the existing Act in regard to the rate of sickness benefit payable to insured persons, other than voluntary contributors, who were aged fifty years and upwards on entry into insurance within one year of the commencement of the Act (July 15, 1912). The rate of benefit is to be the level rate of 10s. a week for men and 7s. 6d. a week for women, reducible only on account of arrears of contributions or in the future as the result of a valuation, and is not to be reduced progressively with the increase of age as under the existing enactment.

This is a very commendable proposal. It will, of course, give satisfaction to the old persons who would only have been entitled to reduced benefit. It will also give satisfaction to the officials of approved societies, as it will materially simplify the accountancy. And it will help the officials at the Insurance Commission Offices, as it will no longer be so imperative to investigate the rates of benefit paid. Such an alteration will involve a considerable amount of labour to bring it into operation. The initial reserves will have to be recalculated, and office records and insurance books will require alteration. For this reason time will have to be allowed to enable the proposal to be brought into effect, and it is provided that the Insurance Commissioners may appoint a date not later than January 14, 1914. The Insurance Commissioners are also left to draw up the regulations which they may consider necessary to enable the transition of rates to be made.

By the addition to the reserves already referred to the amount of the initial deficiency will be increased, and the Chancellor of the Exchequer stated that it was estimated that the period over which this deficiency is to be liquidated will be extended by two years, so that the full value of the contributions paid by the insured persons at age sixteen at entry will not be obtained until at least twenty years have elapsed from the commencement of the Act.

### Answers to Correspondents.

NOTE.—Questions and Correspondence on all doubtful points are invited, and should be addressed to the Editor, THE HOSPITAL, 28, 29 Southampton Street, Strand, London, W.C., and marked "Insurance."

